# An Epidemiological Study on the Effectiveness of Nasturtium Herb and Horseradish Root (Angocin^®^ *Anti-Infekt N*) as well as Other Phytopharmaceuticals, Synthetic Products, and Antibiotics on the Course of Acute Upper Respiratory Tract Infection (aURTI)

**DOI:** 10.3390/antibiotics15040336

**Published:** 2026-03-26

**Authors:** Nina Kassner, Meinolf Wonnemann, Yvonne Ziegler, Karel Kostev

**Affiliations:** 1 Repha GmbH Biologische Arzneimittel, 30855 Langenhagen, Germany; 2 University of Marburg, University Clinic, 35043 Marburg, Germany; 3 IQVIA, Epidemiology, 60549 Frankfurt, Germany

**Keywords:** Angocin^®^ *Anti-Infekt N*, real-world evidence, upper respiratory tract infection, rhinosinusitis, rhinitis, sinusitis, antibiotic, mucolytic, phytotherapy

## Abstract

**Background:** The goal of this study was to evaluate whether medical recommendation of Angocin^®^ *Anti-Infekt N* (hereafter referred to as Angocin^®^) on the day of diagnosis of an acute upper respiratory tract infection (aURTI) or acute sinusitis (AS) is negatively associated with a recurrence of these diagnoses, incidence of antibiotic prescriptions, incidence of chronic sinusitis, nasal polyps, or sick leave duration. **Methods:** This retrospective cohort study utilized the IQVIA^TM^ Disease Analyzer database and included patients by general practitioners with at least one diagnosis of aURTI or AS from 2005 to 2024 and a prescription of Angocin^®^, nasal medications (xylometazoline, oxymetazoline) and mucolytics (ambroxol or acetylcysteine), other phytopharmaceutical drugs, or antibiotics on the day of diagnosis. Patients who received Angocin^®^ were matched separately to each of the three comparison cohorts in a 1:5 ratio using a nearest-neighbor propensity score approach. The relationship between Angocin^®^ prescription and the risks of a recurrence, subsequent antibiotic use or progression to chronic disease was then estimated with Cox proportional hazard models. To examine whether Angocin^®^ exposure was associated with the length of sick leave, univariable conditional logistic regression was applied. **Results:** A total of 3501 Angocin^®^ patients and 17,505 patients in each further cohort were investigated. Angocin^®^ prescription was associated with a significantly lower incidence of a newly diagnosed aURTI/AS as compared to other phytopharmaceuticals (Hazard ratio (HR): 0.78; 95% confidence interval (CI): 0.68–0.86), nasal medications and mucolytics (HR: 0.79; 95% CI: 0.71–0.88), or antibiotics (HR: 0.85; 95% CI: 0.77–0.95). In addition, there was a significantly lower incidence of subsequent further prescriptions of antibiotics when compared to other phytopharmaceuticals (HR: 0.92; 95% CI: 0.82–0.99), nasal medications and mucolytics (HR: 0.87 (95%; CI: 0.80–0.95), or antibiotics (HR: 0.62; 95% CI: 0.57–0.67). Furthermore, Angocin^®^ was associated with the most advantageous pattern of work absence across all time periods examined. **Conclusions:** Considering the limitations of the study, the results cast a positive light on Angocin^®^ prescription in the management of aURTI/AS, particularly with regard to recurrence rates, subsequent antibiotic prescriptions, and sick leave duration.

## 1. Introduction

The upper respiratory tract, consisting of the nasal passages, pharynx, larynx, and proximal trachea, is a primary portal of entry for a myriad of respiratory pathogens. Acute upper respiratory tract infections (aURTIs) encompass a spectrum of infectious diseases affecting these anatomical regions, including the common cold, rhinitis, sinusitis, pharyngitis, and laryngitis [1]. Common symptoms include nasal congestion, anterior and posterior rhinorrhea, sore throat, cough, and fever [2]. Acute sinusitis (AS) is often also described as acute rhinosinusitis (ARS) since inflammation of the sinuses rarely occurs without concurrent inflammation of the nasal mucosa [3]. Despite their often self-limiting nature, aURTIs can lead to severe complications, particularly in vulnerable populations such as the elderly, infants, and immunocompromised individuals. aURTIs are usually caused by viruses and rarely by bacterial pathogens [4]. Up to 2% of patients with viral ARS also develop a bacterial infection [3]. Antibiotics are not advised in the absence of evidence for a bacterial infection, yet they continue to be prescribed at high rates for patients with acute aURTI/AS in primary-care settings [5]. Point-of-care laboratory tests that could differentiate between viral and bacterial infections are used only infrequently in routine clinical practice, largely because their results are not available for several days and they generate additional costs [6].

Bacterial resistance burden has increased in recent years, mainly due to inappropriate antibiotic use and has become an urgent public health concern. Therefore, antibiotic stewardship for aURTI focuses on reducing the unnecessary, inappropriate, and excessive prescription of antibiotics for conditions that are mostly viral and self-limiting (e.g., common cold, rhinosinusitis) [7]. The goal is to “spare” conventional, broad-spectrum antibiotics to reduce antimicrobial resistance, adverse drug reactions, and costs. Differential diagnosis to distinguish between viral and bacterial etiologies and non-antibiotic treatment strategies are essential to mitigate antibiotic overuse [8,9].

The treatment of aURTI focuses mainly on managing symptoms (symptomatic treatment). Single or combination use of synthetic nasal decongestants (e.g., xylometazoline) and NSAIDs is often used to combat symptoms of aURTI/AS. Although not mentioned in the treatment guidelines of aURTI/AS, over-the-counter (OTC) mucolytics such as ambroxol and acetylcysteine dominate the common cold OTC market in Germany, accounting for an impressive market share of up to 47.4% [10].

Phytopharmaceutical drugs, derived from plant-based sources, have been a focal point of interest in recent years due to their diverse bioactive compounds and are often used for the treatment of aURTI. These compounds often exhibit anti-inflammatory, antimicrobial, antiviral, secretolytic, and immunomodulatory properties, making them valuable for addressing aURTIs. Unlike synthetic medications, phytopharmaceuticals offer the advantage of an integrated approach, targeting multiple facets of the disease while minimizing adverse effects [11,12].

Among these, Angocin^®^ *Anti-Infekt N* (referred to hereafter as Angocin^®^) demonstrated effectiveness in the therapy of acute sinusitis/aURTI. Angocin^®^ is a phytopharmaceutical drug composed of natural active ingredients, namely nasturtium (*Tropaeolum majus*) and horseradish (*Armoracia rusticana*) powder. The efficacy of Angocin^®^ in aURTI/AS is attributed to its multi-faceted mechanisms of action. Both plants are known to have antimicrobial properties mainly mediated by their content of isothiocyanates, also known as mustard oils [13,14]. The combination of the two plant components has been shown to produce synergistic antibacterial activity *in vitro*. Ongoing research is examining the antiviral potential of Angocin^®^ against both enveloped and non-enveloped respiratory viruses. To date, antiviral effects have been demonstrated for related isothiocyanates—such as L-sulforaphane—against common pathogens like the respiratory syncytial virus (RSV) [15] and most recently against the SARS-CoV-2 virus [16]. Furthermore, both plants influence relevant signaling pathways that are responsible for the release of inflammatory mediators [17,18]. The plants are also used in traditional medicine as an expectorant [19].

Many studies with Angocin^®^ have been conducted to assess its effectiveness in the treatment of respiratory tract infections [17,20]. Goos et al. performed two prospective studies investigating the effectiveness and safety profile of Angocin^®^ in acute infections, including AS in comparison with other treatments in adults (*n* = 1654) and children (*n* = 858). The effectiveness of a therapy with Angocin^®^ for AS was comparable to the treatment with standard antibiotics; however, it had an advantageous safety profile [21,22]. Furthermore, Albrecht et al. conducted a randomized, double-blinded, placebo-controlled, multicenter clinical trial including 380 patients with ARS. Responder rates assessed by an ARS symptom score were significantly higher for patients receiving Angocin^®^ compared to placebo [23]. A further randomized, placebo-controlled clinical study demonstrated the effectiveness of Angocin^®^ in the management of acute bronchitis [24]. Another placebo-controlled double-blind study showed that administration of Angocin^®^ significantly reduced the frequency of cold episodes by 48% (*p* = 0.0171) during a 12-week treatment period compared to placebo [25].

However, there is a lack of real-world evidence for the effectiveness of Angocin^®^ compared to a standard antibiotic therapy as well as other frequently used drugs (phytopharmaceutical and synthetic drugs) in patients with aURTI/AS. The goal of this study was to determine whether recommending Angocin^®^ at the time of an aURTI/AS diagnosis is associated with a lower likelihood of recurrent aURTI/AS episodes, subsequent antibiotic prescribing, the development of chronic sinusitis or nasal polyps, and sick leave.

## 2. Methods

### 2.1. Data Source

This retrospective cohort study utilized the IQVIA^TM^ (formerly known as IMS Health) Disease Analyzer database. This database is a large electronic health record system that compiles information on patient demographics, diagnoses, and prescribed medications. It draws on data provided by office-based physicians in Germany, including both general practitioners and medical specialists. The database contains records from more than 11 million individuals over the years 2005 to 2024. Participating practices are distributed across eight major regions of Germany, ensuring broad geographic representation. All data collection and processing procedures comply with German data protection regulations. The sampling strategy employed in Germany for selecting physicians’ practices is considered suitable to yield a representative database of both general and specialized practices, as previously demonstrated by Rathmann et al. [26]. Widely recognized and employed, this database has been instrumental in numerous published studies focused on acute upper respiratory tract infections (aURTIs) and sinusitis, as evidenced by works such as those by Bittner et al. [27] and Kern et al. [6].

### 2.2. Study Population

Patients were eligible for inclusion if they were treated in outpatient primary care in Germany and had at least one documented diagnosis of an acute upper respiratory tract infection (aURTI) including sinusitis according to the “International Statistical Classification of Diseases and Related Health Problems” ICD code (ICD-10: J01: Acute sinusitis, J06: Acute upper respiratory infections of multiple and unspecified sites) from January 2005 to December 2024. Patients with a first documentation of one of the upper respiratory tract infections were categorized into one of four cohorts according to the “Anatomical Therapeutic Chemical” (ATC) classification system based on the prescription of defined therapy on the day of the diagnosis:

(1) Angocin^®^ *Anti-Infekt N* (R05XP50) on the day of the diagnosis;
(2) nasal medications (xylometazoline, dexpanthenol + xylometazoline, oxymetazoline; R01AA07, R01AB06) or mucolytics (ambroxol (R05CB06) or acetylcysteine (R05CB01) on the day of the diagnosis;
(3) defined phytopharmaceutical drugs (myrtole (R05CP59), cineole (R05CA13), eucalyptus globulus + pinus sylvestris (R04AP30), combination of primrose flowers, gentian root, sorrel herb, elder flowers and vervain herb (R01BP30), and combination of chamomile, marshmallow, horsetail, yarrow, walnut, dandelion and oak (R02AP30)) on the day of the diagnosis;
(4) antibiotics (ATC: J01) on the day of the diagnosis.

The Anatomical Therapeutic Chemical (ATC) code is a unique identifier that classifies each medication according to the organ system and pharmacologic action, as defined by the World Health Organization. Patients were excluded if they had received the study medication or any other cold-related treatments (ATC R01, R04, R05) within the 30 days preceding the index date, if more than one study therapy was prescribed on the index date, if information on age or sex was missing, or if a diagnosis of chronic sinusitis (ICD-10: J32) or nasal polyps (ICD-10: J33) had been recorded before or on the index date.

### 2.3. Descriptive and Statistical Analyses

To minimize selection bias and reduce the influence of confounding variables, a matched-pairs design was applied. Individuals receiving Angocin^®^ were matched separately to each of the three comparison cohorts in a 1:5 ratio using a nearest-neighbor propensity score procedure based on age, sex, health insurance type, and the Charlson Comorbidity Index (CCI). The CCI reflects the burden of comorbidity by summing 17 ICD-10 defined conditions, yielding a score from 0 to 17, with higher values indicating greater morbidity [28]. Standardized mean difference (SMD) is widely used to assess whether covariates are balanced between treatment groups after matching. In this study, an SMD below 0.25 was taken as evidence that the matching procedure achieved an acceptable level of covariate balance [29].

All recurrence analyses were performed over a unified 12-month follow-up period (1–365 days). Only renewed diagnoses accompanied by a relevant prescription (Angocin^®^, antibiotics, nasal medications/mucolytics, other phytopharmaceutical preparations) were counted as events. For each cohort comparison, Kaplan–Meier curves were generated to depict cumulative incidence of renewed aURTI/AS. Associations between a treatment cohort and recurrence were evaluated using univariable conditional Cox regression, performed for the total matched cohort and men and women separately.

Antibiotic prescribing was assessed over 1–365 days after the index date. Kaplan–Meier curves were used to estimate cumulative proportions of patients receiving at least one subsequent antibiotic prescription within this period. Associations between index therapy and receipt of a new antibiotic prescription were evaluated using univariable conditional Cox regression, stratified by sex and age groups as described above.

The proportion of patients who received a first diagnosis of chronic sinusitis or nasal polyps (ICD 10: J32, J33) within three years after the index date was compared between the Angocin^®^ group and each comparison cohort using conditional Cox regression models. In this context, the hazard reflects the event rate within a given group, while the hazard ratio represents the ratio of these hazards and indicates how much more or less frequent the event occurs in one group compared to the other.

Sick leave documented within 1–30 days after the index date was evaluated. The presence and duration of sick leave (>3 days, >7 days, ≥10 days) were assessed for the full matched cohorts. Associations between index therapy and sick-leave duration categories were analyzed using univariable conditional logistic regression.

All regression analyses described previously were repeated to compare cohorts 2 (nasal medications/mucolytics) and 3 (other predefined phytopharmaceutical drugs) with cohort 4 (antibiotics). In addition, all regression models were repeated in sensitivity analyses, adjusting for the number of consultations within 12 months prior to the index date, the frequency of prior aURTI/AS diagnoses within the same period, GP practice ID (fixed effect), and inclusion year.

*p*-values of <0.05 were considered statistically significant in all analyses. SAS Vers. 9.4 (SAS Institute, Cary, NC, USA) was used to conduct the analyses.

## 3. Results

### 3.1. Baseline Characteristics of Study Patients

**Figure 1:**
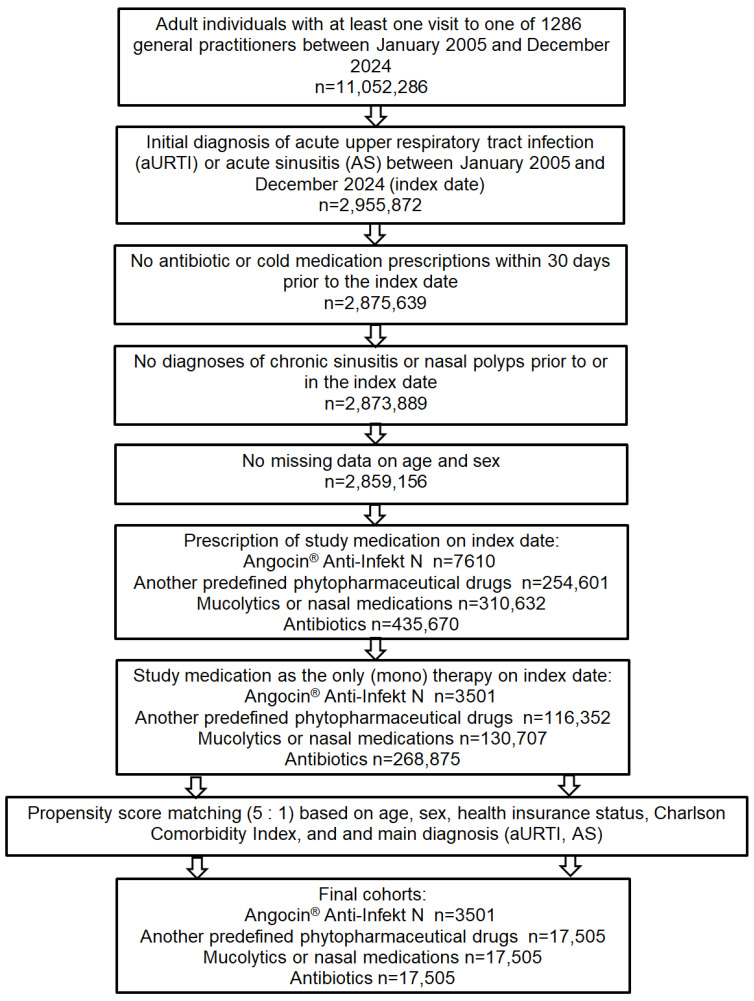
Selection of study patients.

Of 2,859,156 patients diagnosed with an aURTI or AS and fulfilling all inclusion criteria, Angocin^®^ was prescribed to 3501 patients as the only (mono) therapy on index date, nasal medications/mucolytics to 130,707, other predefined phytopharmaceuticals to 116,352, and antibiotics to 268,875 patients, respectively. After 1:5 propensity score matching, 3501 Angocin^®^ patients and 17,505 patients in each of the three comparison cohorts were available for analysis (Figure 1).

Across all four matched cohorts, patient characteristics were highly similar (Table 1). The mean age was approximately 41 years, with ~59% female, ~13–14% privately insured, and ~6% diagnosed with sinusitis at index date. The Charlson Comorbidity Index was consistently 0, indicating a predominantly healthy study population. Standardized mean differences remained well below 0.25 (maximum = 0.016) for all matched variables, confirming adequate balance.

### 3.2. Recurrence of the aURTI/AS

**Figure 2:**
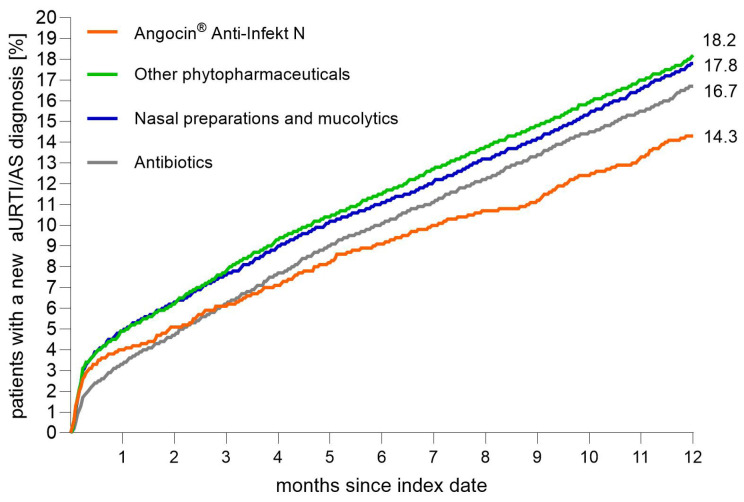
Cumulative 12-month-incidence of a new aURTI/AS diagnosis after the index date (Kaplan–Meier curves).

A newly diagnosed aURTI/AS was documented in 14.3% of patients with Angocin^®^ prescription, 18.2% of patients with prescription of other phytopharmaceuticals, 17.8% of patients with nasal medications/mucolytics, and 16.7% of patients with antibiotic prescription within 365 days after the index date (Figure 2).

Angocin^®^ was associated with a significantly lower probability of recurrence compared with other phytopharmaceuticals in the total cohort, with a hazard ratio (HR) of 0.78 (95% CI: 0.68–0.86). A similarly lower recurrence probability was observed when Angocin^®^ was compared with nasal medications/mucolytics, yielding an HR of 0.79 (95% CI: 0.71–0.88).

Compared with an antibiotic therapy, Angocin^®^ showed an HR of 0.85 (95% CI: 0.77–0.95), indicating a more modest but statistically significant association with fewer recurrences. Analyses stratified by sex and age showed consistent patterns. For example, among women, Angocin^®^ was associated with lower recurrence compared with other phytopharmaceuticals (HR 0.76; 95% CI: 0.68–0.89) and with nasal medications/mucolytics (HR 0.79; 95% CI: 0.69–0.91). In men, the associations were similar, with HRs of 0.76 (95% CI: 0.64–0.90) versus other phytopharmaceuticals and 0.78 (95% CI: 0.66–0.93) versus nasal medications/mucolytics.

The regression models also quantified associations for the other therapy groups relative to antibiotics. Mucolytics and nasal preparations were associated with a higher recurrence probability than antibiotics, with an HR of 1.08 (95% CI: 1.02–1.15) in the total cohort, while other phytopharmaceuticals were likewise associated with a higher recurrence probability relative to antibiotics, with an HR of 1.11 (95% CI: 1.05–1.17). These patterns were generally consistent across sexes (Table 2).

### 3.3. Antibiotic Prescription After Index Date

**Figure 3:**
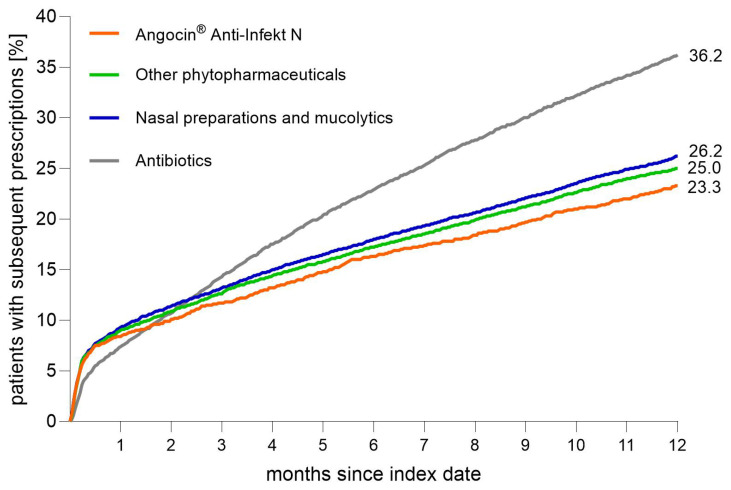
Cumulative 12-month incidence of patients receiving a new (further) antibiotic prescription after index date and initial prescription (Kaplan–Meier curves).

During the 12-month follow-up, new antibiotic prescriptions occurred in 23.3% of patients who had received Angocin^®^, compared with 25.0% among those receiving other phytopharmaceuticals, 26.2% among those treated with nasal medications/mucolytics, and 36.2% in patients treated initially with antibiotics (Figure 3).

The regression analyses showed that Angocin^®^ was associated with a lower probability of receiving an antibiotic prescription during follow-up when compared with other phytopharmaceuticals, with a hazard ratio of 0.92 (95% CI: 0.82–0.99). A similar association was observed in comparison with nasal medications/mucolytics, with an HR of 0.87 (95% CI: 0.80–0.95). When compared with an antibiotic therapy on the index day, Angocin^®^ was associated with a clearly reduced 12-month probability of further antibiotic prescribing, with an HR of 0.62 (95% CI: 0.57–0.67).

The comparator therapies also showed distinct associations relative to antibiotic treatment. Mucolytics and nasal preparations were associated with a lower probability of subsequent antibiotic use compared with initial antibiotic therapy, with an HR of 0.71 (95% CI: 0.68–0.74). Other phytopharmaceuticals showed a comparable association, with an HR of 0.67 (95% CI: 0.64–0.70). Thus, among all four therapy strategies, patients initially treated with antibiotics had the highest likelihood of receiving additional antibiotics during the 12-month follow-up, whereas Angocin^®^ showed the lowest (Table 3).

Across subgroups, these patterns persisted. For example, in adults aged 31–45 years, Angocin^®^ was associated with a lower probability of antibiotic prescription compared with other phytopharmaceuticals (HR 0.84; 95% CI: 0.72–0.98) and with mucolytics/nasal medications (HR 0.82; 95% CI: 0.70–0.96). The association relative to antibiotics remained, with an HR of 0.55 (95% CI: 0.47–0.64).

### 3.4. Incidence of Chronic Sinusitis and Nasal Polyps

The incidence of chronic sinusitis or nasal polyps was 22.6 cases per 1000 person-years among Angocin^®^ patients, 24.3 cases among patients with prescription of other phytopharmaceuticals, 20.9 cases among patients with prescription of nasal medications or mucolytics, and 22.3 among antibiotic patients. Angocin^®^ demonstrated neither a statistically significant association with higher or lower incidences compared to other phytopharmaceuticals, with an HR of 0.93 (95% CI: 0.83–1.05), nor compared to nasal medications/mucolytics (HR 1.05; 95% CI: 0.93–1.19), or antibiotics (HR 0.96; 95% CI: 0.85–1.09) (Table 4).

Among the comparator cohorts, nasal medications/mucolytics did not differ from antibiotics (HR 1.03; 95% CI: 0.97–1.11). The only significant association in these analyses was observed between other phytopharmaceuticals and antibiotic therapy, as other phytopharmaceuticals were associated with a slightly lower incidence of chronic sinusitis or nasal polyps, with an HR of 0.92 (95% CI: 0.86–0.98). Overall, none of the therapies, including Angocin^®^, showed evidence of a clinically meaningful association with long-term chronic sinusitis or nasal polyp development (Table 4).

### 3.5. Sick Leave Associated with aURTI/AS

**Figure 4:**
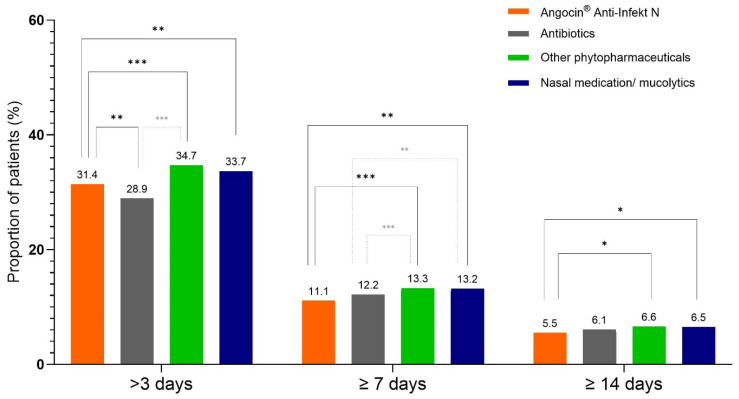
Proportion of patients with a sick leave duration of >3, >7 and ≥10 days. Significance values represent the results of the associations given in Table 5. * *p* < 0.05, ** *p* < 0.01; *** *p* < 0.001.

Among patients with documented sick leave, the Angocin^®^ cohort consistently showed the smallest proportions of prolonged sick leave durations, while the antibiotic cohort showed the highest proportions (Table 5/Figure 4). Angocin^®^ was associated with a lower probability of sick leave exceeding three days compared with phytopharmaceuticals, with an odds ratio (OR) of 0.86 (95% CI: 0.80–0.93). The association with nasal medications/mucolytics was similar (OR 0.90; 95% CI: 0.83–0.98). When compared with antibiotics, Angocin^®^ was associated with a higher probability of taking more than three sick leave days (OR 1.13; 95% CI: 1.04–1.22).

For sick leave periods longer than seven days, Angocin^®^ was associated with a lower probability than other phytopharmaceuticals (OR 0.81; 95% CI: 0.73–0.91) and mucolytics/nasal preparations (OR 0.83; 95% CI: 0.74–0.93). The comparison with antibiotics showed a slight trend towards a lower probability, although the 95% CI included unity (OR 0.91; 95% CI: 0.81–1.02) and therefore was not significant.

For sick leave durations of at least ten days, Angocin^®^ showed lower probabilities compared with other phytopharmaceuticals (OR 0.82; 95% CI: 0.71–0.97) and a similar association relative to nasal medications/mucolytics (OR 0.83; 95% CI: 0.71–0.97). The comparison between Angocin^®^ and antibiotics again suggested a slight trend towards lower probability, though without statistical significance (OR 0.89; 95% CI: 0.76–1.04).

### 3.6. Sensitivity Analyses

Extended regression models adjusted for the number of consultations within 12 months prior to the index date, the frequency of prior aURTI/AS diagnoses within the same period, GP practice ID (fixed effect), and inclusion year yielded results consistent with the primary analyses. For example, Angocin^®^ was associated with a significantly lower probability of recurrence compared with other phytopharmaceuticals (HR 0.77; 95% CI 0.69–0.86), nasal medications/mucolytics (HR 0.79; 95% CI 0.71–0.88), and antibiotic therapy (HR 0.86; 95% CI 0.78–0.96). Additional regression analyses also showed that Angocin^®^ was associated with a lower probability of receiving an antibiotic prescription during follow-up compared with other phytopharmaceuticals (HR 0.92; 95% CI 0.84–0.99), nasal medications/mucolytics (HR 0.88; 95% CI 0.81–0.96), and antibiotic therapy (HR 0.63; 95% CI 0.58–0.68).

## 4. Discussion

This large real-world analysis provides a comprehensive evaluation of Angocin^®^ relative to commonly used therapeutic alternatives for acute upper respiratory tract infections (aURTI) and acute sinusitis (AS). The robustness of our findings is supported by the large, representative primary-care population and the optimal covariate balance achieved through propensity score matching, which minimizes selection bias. The consistency of associations across multiple outcomes—recurrence, subsequent antibiotic use, and sick -leave duration—further underscores the reliability of the observed patterns. Importantly, the results align well with existing clinical evidence on Angocin^®^, suggesting that its multi-target pharmacological profile may translate into meaningful real-world benefits.

A consistent finding across the analyses is that Angocin^®^ was associated with a more favorable 12-month course after the index date than the other non-antibiotic therapies. Patients receiving Angocin^®^ demonstrated a lower probability of returning with a renewed, prescription-associated diagnosis of aURTI/AS compared with those treated with other phytopharmaceuticals or with nasal medications and mucolytics. These associations were evident not only in the overall population but also across sex, suggesting that the pattern is robust and not confined to a particular demographic subset. Relative to antibiotic therapy, Angocin^®^ showed lower recurrence patterns over the year, while the two non-antibiotic comparator groups were associated with comparatively less advantageous recurrence profiles.

A previous investigation demonstrated that the continuous intake of Angocin^®^ over 3 months can lower the likelihood of subsequent respiratory tract infections. In this randomized, prospective, placebo-controlled, double-blind study, 344 participants were allocated to three treatment arms. Over a 12-week period, individuals received either 3 × 2 Angocin^®^ tablets daily (group 1), 2 × 2 Angocin^®^ tablets plus 1 × 2 placebo tablets (group 2), or 3 × 2 placebo tablets (placebo group). The primary study endpoint was the number of cold episodes occurring during the three-month treatment phase. In the first group, the re-infection rate was 13.3%, in group 2 18.4%, and in the placebo group 25.6%, respectively. A statistically significant and clinically relevant difference in favor of the high dosage (3 × 2 tablets) versus placebo (*p* = 0.0171) showed almost 50% fewer colds [25]. This outcome is in line with the results of this retrospective study. The sustained effect of Angocin^®^ over the 365-day period in this study could also be attributed to the fact that patients who were satisfied with the initial effect of the product used it repeatedly without further medical advice or prescription.

Besides the known antibacterial [13,14], anti-inflammatory [17,18,20] and antiviral effect [16] of the active compounds, the isothiocyanates (ITC) in Angocin^®^, it can be postulated that treatment effects described above might be due to an activation or upregulation of parts of the innate immune system. Evidence from a human consumption study showed that intake of ITC-releasing nasturtium by healthy volunteers led to a significant rise in human beta-defensin-1 (hBD-1) levels in urine and exhaled breath condensate [30]. HBD-1 as an antimicrobial peptide, is predominantly produced on epithelial cells of the respiratory and urinary tract and is part of the innate immune system responsible for the elimination of viruses, bacteria and fungi.

In this study, Angocin^®^ was also consistently associated with the lowest likelihood of subsequent antibiotic use across all comparison groups. But, a comparison with antibiotics could always be critically questioned, as patients who receive an antibiotic on the day of diagnosis are often presumed to be more severely ill than those treated with phytopharmaceuticals such as Angocin^®^ or other over-the-counter medications. It is generally estimated that 2 to 10% of cases of ARS are caused by bacteria, particularly among patients with underlying health conditions [31]. However, Kern & Kostev reported that in Germany over 50% of adults with acute rhinosinusitis (ARS) were prescribed antibiotics [5], although most of the guidelines for the treatment of ARS recommend avoiding antibiotics [4,32]. This is particularly noteworthy given the ongoing public health emphasis on antibiotic stewardship. That all non-antibiotic treatments—including Angocin^®^—were associated with fewer follow-up antibiotic prescriptions than initial antibiotic therapy highlights a broader pattern: therapeutic strategies that avoid antibiotics at the index visit tend to be associated with a reduced long-term need for antimicrobial agents. Also, another study showed that prescriptions of phytopharmaceuticals significantly reduced the likelihood of antibiotic use [9]. Antibiotic use may also impair aspects of immune memory, potentially leaving patients more vulnerable to subsequent viral infections [33]. In this context, Angocin^®^ displays a favorable profile, aligning with the growing interest in phytopharmaceutical options that can support recovery while mitigating unnecessary antibiotic exposure.

Furthermore, Angocin^®^ was associated with an overall advantageous pattern of sick leave across the time periods examined. Patients receiving this therapy exhibited fewer prolonged episodes of incapacity for work compared with those treated with other phytopharmaceuticals, nasal medications/mucolytics, or antibiotics. However, treatment with Angocin^®^ revealed higher rates of short-term sick leave of up to 3 days in comparison to antibiotic treatment in this analysis. The groups of other phytopharmaceuticals and nasal medications/mucolytics showed a similar pattern, even for up to 7 days. These observations likely reflect differences in symptom resolution, functional recovery, or help-seeking behavior, although the database does not permit deeper exploration of these mechanisms. Since sick leave might also be a sign of disease severity, it cannot be related to the treatment efficacy but likely mirrors the pretreatment status of the patient at the index date. In general, sick leave outcomes may also reflect socioeconomic or occupational differences rather than the effect of treatment, so the findings on sick leave duration should not be overinterpreted. From a health-economic and societal standpoint, however, the overall pattern underscores the potential of Angocin^®^ to contribute to shorter functional impairment in real-world settings.

In contrast, long-term structural outcomes such as chronic sinusitis or nasal polyps showed no meaningful differences between Angocin^®^ and any comparator therapy. All therapies—including antibiotics—demonstrated comparable rates of these diagnoses, indicating that such chronic conditions likely evolve largely independently of the initial therapeutic pathway selected for an acute episode.

In the present study, Angocin^®^ was compared with other commonly used therapies. One of these is a combined cohort of nasal medications and mucolytics. In 2018, Cazan et al. published a review assessing the effectiveness and safety of ambroxol for treating airway diseases in adults. Several clinical studies cited in this review demonstrated that ambroxol effectively alleviates respiratory symptoms and reduces the likelihood of acute exacerbations [34]. However, there are no large real-world analyses on mucolytic effectiveness in the adult population. Furthermore, although in Germany ambroxol and acetyl-cysteine are the commonly used expectorants, there is limited data supporting their efficacy in treating patients with aURTI [35].

Another study cohort of this analysis contains different essential oils and phytopharmaceutical combinations. For example, the combination of primrose flowers, gentian root, sorrel herb, elder flowers and vervain herb was already investigated in a large study using real-world data and even the same data source, like in the present study [36]. In this study, the prescription was associated with a lower probability of longer sick leaves ≥7 days and less frequent visits to a general practitioner or an ears, nose and throat specialist in the first 30 days after the index date when compared to antibiotics, but not when compared to nasal medications without corticosteroids. As the study group was not structured in the same way as in our study, no direct comparison of the results can be made.

Taken together, the results form a coherent pattern: Angocin^®^ was associated with favorable outcomes in domains of recurrence of aURTI/AS, necessity of subsequent antibiotic use, and sick leave, while demonstrating equivalence with other therapies regarding long-term structural or chronic sinonasal outcomes. Although observational research cannot account for all potential sources of confounding, the consistency of associations across multiple endpoints and matched comparison cohorts strengthens confidence in the overall interpretation.

The authors are well aware of the limitations presented here and the associated restrictions on interpretation. In addition, great importance was attached to presenting the employment relationships and funding in a transparent manner. The authors would very much welcome the opportunity to verify the effects and findings presented here in a less limited setting or in the context of clinical studies in the future.

## 5. Conclusions

In conclusion, Angocin^®^ appears to have a clear long-term negative association regarding the aURTI/AS recurrence rate compared to other phytopharmaceuticals and nasal medications/mucolytics or antibiotics. In addition, Angocin^®^ appears to be negatively associated with new subsequent antibiotic prescriptions compared to other phytopharmaceuticals, nasal medications and mucolytics, or antibiotics. Finally, it shows an advantageous association with sick leave duration compared to other treatments.

## 6. Limitations

The findings of this study should be interpreted with some caution, as several important study- and database-related limitations need to be acknowledged. First, the database does not record the use of herbal medicines purchased over the counter. In Germany, Angocin^®^ can be obtained without a physician’s prescription, meaning this use remains undocumented. Second, diagnoses of aURTI and AS were based solely on ICD codes entered by general practitioners, which do not allow for differentiation between viral and bacterial infections. Therefore, microbial etiology and the reason for an aURTI cannot be deduced from the underlying data set. Moreover, association results should be handled with care as the underlying IQVIA^TM^ Disease Analyzer database does not allow for an adjustment between different severity stages of diseases or a general symptom burden. The database was not designed according to the principles of a medical registry study. Since the general prescribing behavior of doctors is not clear, it cannot be estimated whether patients with a certain severity were assigned to a particular therapy. There might indeed be underlying preferences for either antibiotic or non-antibiotic treatment, as well as other unmeasured factors that influence the therapeutic choice in cases of infection or reinfection. In addition, the analysis cannot account for individual patient preferences that may guide treatment decisions. However, this assumption can at least partially be refuted. Ehrenberg and colleagues examined determinants of phytopharmaceutical prescribing in German outpatient care and found that a practice-level preference for phytotherapeutics was associated with a six-fold higher likelihood of receiving such a prescription, independent of diagnosis or patient characteristics [37].

Third, the diagnosis code ICD-10 J06 refers to acute upper respiratory infections of multiple and unspecified sites and can be based on different symptoms like sneezing, stuffy or runny nose, sore throat, coughing, or fever. The database does not include data on symptom severity, even though specific symptoms may influence the choice of therapy. In addition, data on socioeconomic status and lifestyle-related risk factors are not available, limiting the ability to account for these potentially relevant determinants. Finally, due to the study design, only associations, rather than causal relationships, can be inferred.

## Figures and Tables

**Table 1:** Basic characteristics of study patients.

Variable	Angocin^®^ (N, %)	Antibiotics (N, %)	SMD	Other Phytopharmaceutical Drug (N, %)	SMD	Mucolytic or Nasal Medication (N, %)	SMD
N	3501	17,505		17,505		17,505	
Age (mean, SD)	41.0 (16.3)	41.0 (16.2)	0.000	41.1 (16.3)	−0.002	41.2 (16.5)	−0.009
<18 years (%)	85 (2.4)	427 (2.4)		430 (2.5)		444 (2.5)	
18-30 years (%)	1014 (29.0)	5067 (29.0)		5060 (28.9)		5050 (28.9)	
31–45 years (%)	1111 (31.7)	5550 (31.7)		5556 (31.7)		5516 (31.5)	
46–65 years (%)	1013 (29.0)	5071 (29.0)		5065 (28.9)		5046 (28.8)	
>65 years (%)	278 (7.9)	1390 (7.9)		1394 (8.0)		1449 (8.3)	
Sex: female (%)	2087 (59.6)	10,403 (59.4)	0.002	10,422 (59.5)	0.001	10,444 (59.7)	−0.001
Private health insurance coverage (%)	459 (13.1)	2272 (13.0)	−0.001	2271 (13.0)	−0.001	2157 (12.3)	−0.008
Sinusitis (%)	206 (5.9)	1030 (5.9)	0.000	1030 (5.9)	0.000	1030 (5.9)	0.000
CCI (median, IQR)	0 (0)	0 (0)	0.008	0 (0)	0.016	0 (0)	0.013

**Table 2:** Association between Angocin^®^ prescription and the probability of a newly diagnosed aURTI/AS within 1–365 days after the index date compared to the other therapies in different patient groups (Hazard ratio, 95% confidence interval). * *p* < 0.05, ** *p* < 0.01; *** *p* < 0.001.

Patient Group	Angocin^®^ vs. Other Phytopharma- ceuticals	Angocin® vs. Nasal Medications/ Mucolytics	Angocin^®^ vs. Antibiotics	Nasal Medications/ Mucolytics vs. Antibiotics	Other Phyto- Pharmaceuticals vs. Antibiotics
Total	0.78 (0.68–0.86) ***	0.79 (0.71–0.88) ***	0.85 (0.77–0.95) **	1.08 (1.02–1.15) **	1.11 (1.05–1.17) ***
Women	0.76 (0.68–0.89) ***	0.79 (0.69–0.91) ***	0.83 (0.73–0.96) **	1.05 (0.98–1.13)	1.08 (1.00–1.16) *
Men	0.76 (0.64–0.90) ***	0.78 (0.66–0.93) **	0.89 (0.75–1.05)	1.13 (1.03–1.24) **	1.16 (1.06–1.27) **

**Table 3:** Association between Angocin^®^ prescription and the probability of a subsequent antibiotic prescription 1–365 days after the index date compared to the other therapies in different patient groups (Hazard ratio, 95% confidence interval). * *p* < 0.05, ** *p* < 0.01; *** *p* < 0.001.

Patient Group	Angocin^®^ vs. Other Phytopharmaceuticals	Angocin^®^ vs. Nasal Medication/Mucolytics	Angocin^®^ vs. Antibiotics	Nasal Medication/Mucolytics vs. Antibiotics	Other Phyto- Pharmaceuticals vs. Antibiotics
Total	0.92 (0.82–0.99) *	0.87 (0.80–0.95) **	0.62 (0.57–0.67) ***	0.71 (0.68–0.74) ***	0.67 (0.64–0.70) ***
Women	0.90 (0.81–1.00)	0.86 (0.77–0.95) **	0.61 (0.55–0.68) ***	0.71 (0.68–0.75) ***	0.68 (0.64–0.71) ***
Men	0.95 (0.85–1.09)	0.90 (0.79–1.04)	0.62 (0.55–0.72) ***	0.69 (0.64–0.74) ***	0.66 (0.61–0.71) ***

**Table 4:** Association between Angocin^®^ prescription and the probability of chronic sinusitis or nasal polyps 12 months after index date compared to other therapies (Hazard ratio, 95% confidence interval).

Comparison	Hazard Ratio (95% CI)	*p*-Value
Angocin^®^ vs. other phytopharmaceuticals	0.93 (0.83–1.05)	0.256
Angocin^®^ vs. nasal medications/mucolytics	1.05 (0.93–1.19)	0.410
Angocin^®^ vs. antibiotics	0.96 (0.85–1.09)	0.547
Nasal medications/mucolytics vs. antibiotics	1.03 (0.97–1.11)	0.337
Other phytopharmaceuticals vs. antibiotics	0.92 (0.86–0.98)	0.012

**Table 5:** Association between Angocin^®^ prescription and the probability of sick leave of more than 3, 7, and 10 days. * *p* < 0.05, ** *p* < 0.01; *** *p* < 0.001.

Sick Leave Duration	Angocin^®^ vs. Other Phyto Pharmaceuticals (OR, 95% CI)	Angocin^®^ vs. Nasal Medications/Mucolytics (OR, 95% CI)	Angocin^®^ vs. Antibiotics (OR, 95% CI)	Other Phyto- Pharmaceuticals vs. Antibiotics (OR, 95% CI)	Nasal Medications/Mucolytics vs. Antibiotics (OR, 95% CI)
N	3501 vs. 17,505	3501 vs. 17,505	3501 vs. 17,505	17,505 vs. 17,505	17,505 vs. 17,505
>3 days	0.86 (0.80–0.93) ***	0.90 (0.83–0.98) **	1.13 (1.04–1.22) **	1.31 (1.25–1.37) ***	1.25 (1.19–1.31)
>7 days	0.81 (0.73–0.91) ***	0.83 (0.74–0.93) **	0.91 (0.81–1.02)	1.11 (1.04–1.18) ***	1.10 (1.03–1.17) **
≥10 days	0.82 (0.71–0.97) *	0.83 (0.71–0.97) *	0.89 (0.76–1.04)	1.08 (0.99–1.18)	1.07 (0.98–1.16)

## Data Availability

The data presented in this study are available on reasonable request from the corresponding author.
